# Clinical Differences in c-Myc Expression in Early-Stage Gastric Neoplasia: A Retrospective Study Based on the WHO Classification

**DOI:** 10.3390/jcm11030544

**Published:** 2022-01-21

**Authors:** Noriyuki Arakawa, Atsushi Irisawa, Kazuyuki Ishida, Takuya Tsunoda, Yoshiko Yamaguchi, Goro Shibukawa, Makoto Eizuka, Shunzo Tokioka, Hiroto Wakabayashi

**Affiliations:** 1 Department of Gastroenterology, Takeda General Hospital, Aizuwakamatsu 965-8585, Japan; tsunotaku@takeda.or.jp (T.T.); shunzo.tokioka.1103@gmail.com (S.T.); wakaba@takeda.or.jp (H.W.); 2 Department of Gastroenterology, Dokkyo Medical University School of Medicine, Mibu 321-0293, Japan; irisawa@dokkyomed.ac.jp; 3 Department of Pathology, Dokkyo Medical University School of Medicine, Mibu 321-0293, Japan; ishida-k@dokkyomed.ac.jp; 4 Department of Pathology, Takeda General Hospital, Aizuwakamatsu 965-8585, Japan; yyamaguchi@takeda.or.jp; 5 Department of Gastroenterology, Aizu Medical Center, Fukushima Medical University, Aizuwakamatsu 969-3492, Japan; goro4649@aol.com; 6 Department of Molecular Diagnostic Pathology, Iwate Medical University School of Medicine, Iwate 028-3694, Japan; m10_makoeizuka@yahoo.co.jp

**Keywords:** gastric cancer, c-Myc, genetic linkage analysis

## Abstract

c-Myc is an oncogene that is dysregulated in various cancers. Early gastric neoplasia with c-Myc expression has been reported as a more malignant lesion. This study clarifies the differences in c-Myc expression in early gastric neoplasia based on the WHO classification. Samples from 100 patients with differentiated-type early gastric neoplasia, who underwent endoscopic submucosal dissection between March 2020 and January 2021, were stained for c-Myc. One hundred lesions were classified as low-grade dysplasia, high-grade dysplasia, or intramucosal adenocarcinoma. The staining intensity and extent were scored. A hierarchical cluster analysis for a clinicopathological analysis among the groups, the chi-square test, Bonferroni correction, and residual analysis were performed. Subgroup one and two consisted of 39 patients; while subgroup three consisted of 22. Significant differences among various characteristics were observed between these subgroups. The frequency of low-grade dysplasia was significantly higher, while that of high-grade dysplasia was significantly lower in subgroup three. The frequency of intramucosal adenocarcinoma was significantly higher in subgroup one. The c-Myc positivity rate was significantly higher in subgroup one compared with that in subgroup three. c-Myc expression distinctly differed in early gastric neoplasia. c-Myc-negative low-grade dysplasia may be separately categorized from c-Myc-positive low-grade dysplasia, high-grade dysplasia, and intramucosal adenocarcinoma.

## 1. Introduction

The use of a genetic analysis to clarify the molecular pathogenesis of gastric cancer has greatly increased in recent years [1]. In Europe and the United States, gastric cancer is diagnosed based on the WHO classification. The intramucosal invasive neoplasia is treated by a mucosectomy or gastrectomy due to the metastatic potential of lesions invading the lamina propria [2]. In Japan, not only an intramucosal adenocarcinoma (IMA), but also low-grade dysplasia (LGD) and high-grade dysplasia (HGD) are targeted for resection. By analyzing the copy number alterations (CNAs) of early-stage gastric cancer, the authors identify several genes that may be related to the early stages of cancer. Among them, a gain in c-Myc (8q24.21) is a genetic abnormality that occurs in the early stage of the disease and may be a driver gene [3]. The CNA analysis of 84 cases of gastric intramucosal epithelial tumors showed that the frequency of 8q gain was increased in HGD and IMA rather than in LGD [4]. It is suggested that the amplification level of c-Myc differs depending on the nuclear and structural atypia. In addition, the gain of a gene has been reported to correlate with an increased protein expression [5].

c-Myc, an oncogene that is dysregulated in various cancers, is involved in carcinogenesis and cancer progression. This gene has also been associated with a variety of biological phenomena, including the promotion of disordered cell growth, neoangiogenesis, metastasis, anaerobic metabolism, and genomic instability [6].

Considering the results of the genetic analysis reported previously, lesions with c-Myc expression in early gastric neoplasia are likely malignant. However, there have been no reports discussing c-Myc expression with a focus on the WHO classification. This study was conducted to clarify the differences in c-Myc expression in early gastric neoplasia based on the WHO classification.

## 2. Materials and Methods

### 2.1. Study Design

This was a retrospective study conducted in a single center and approved by the Clinical Research Ethics Committee of Takeda General Hospital and registered with the University Hospital Medical Information Network (registration number UMIN000044040). Written informed consent was obtained from each patient included in the study, which was performed in accordance with the Declaration of Helsinki. The primary endpoint of the study was hierarchical cluster analysis based on the scores obtained by c-Myc staining to clarify the characteristics of each group. The secondary endpoints of the study were the c-Myc expression rates in early gastric neoplasia based on the WHO classification.

#### 2.1.1. Patients

We evaluated 107 patients who underwent endoscopic submucosal dissection at the Department of Gastroenterology, Takeda General Hospital, between March 2020 and January 2021, and were diagnosed with differentiated-type early gastric neoplasia based on histopathological examination. A total of 100 cases was included, excluding mixed tissue types (cases in which a component of the secondary tissue type accounted for more than 10% of the total, or cases in which the component of the secondary tissue type was small but included poorly differentiated cancer).

#### 2.1.2. Immunohistochemistry

**Figure 1:**
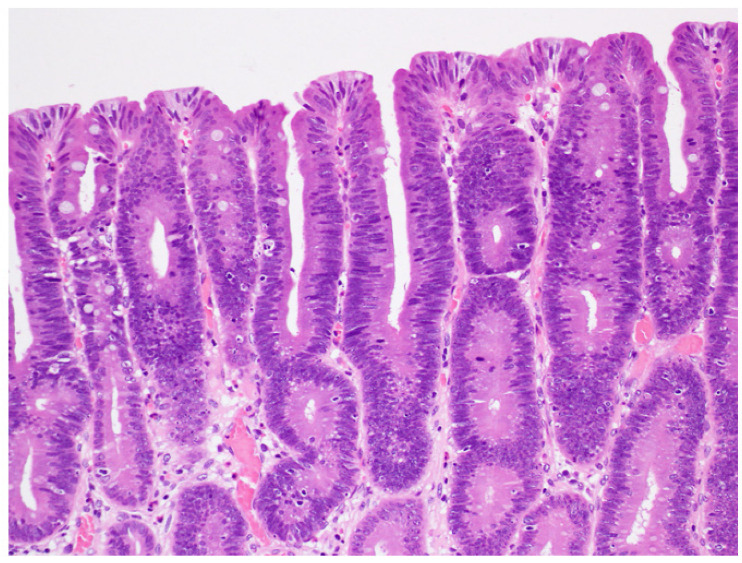
Low-grade dysplasia. Glands are slightly crowed with a regular shape and size. The nuclei are cigar shaped and basally oriented.

**Figure 2:**
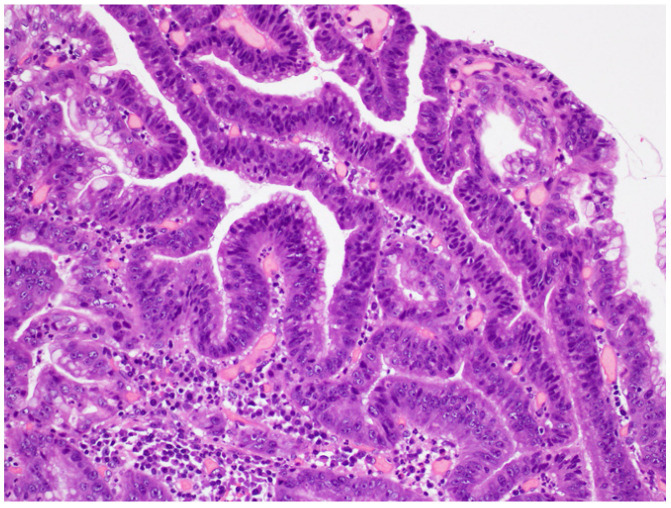
High-grade dysplasia. Glands have a variable size and shape. The nuclei are irregular in shape and size.

**Figure 3:**
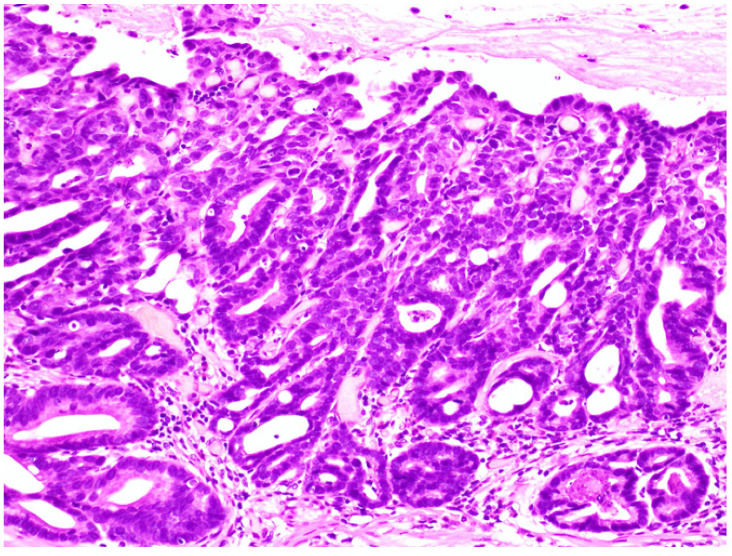
Intramucosal adenocarcinoma. Glands have a complex architecture with irregular branching and glandular anastomosis. Invasion into the lamina propria with no evident desmoplastic reaction.

**Figure 4:**
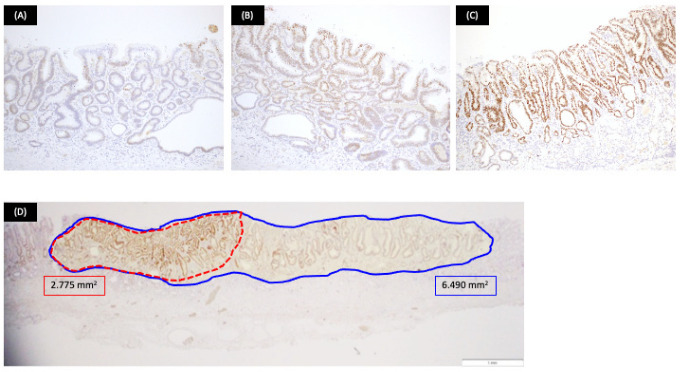
Example of stain interpretation. (**A**): Staining intensity: 1 point (c-Myc; ×40); (**B**): staining intensity: 2 points (c-Myc; ×40); (**C**): staining intensity: 3 points (c-Myc; ×40); (**D**): stained area was measured using the ImageJ software. Red and blue frames show c-Myc positive and gastric neoplasia areas, respectively.

Lesions removed by endoscopic submucosal dissection were fixed in 10% buffered formalin, and the specimens were prepared by total segmentation. The pathological diagnosis was determined following hematoxylin and eosin staining according to the gastric cancer treatment protocol, and the WHO classification was determined [2,7]. One hundred lesions were classified as LGD (Figure 1), HGD (Figure 2), or IMA (Figure 3) using the WHO classification criteria. The WHO classification for intramucosal lesions was used for cases of submucosal invasive cancer. Immunostaining was performed on representative sections following speculum examination. Immunostaining was performed using an automated immunostainer (Histostainer, Nichirei, Tokyo, Japan) and the anti-c-Myc antibody (clone EP121, Nichirei). Staining was evaluated by scoring the intensity and extent of staining (as described below) [8,9]. c-Myc expression was evaluated for nuclear rather than cytoplasmic staining. The staining intensity was classified as negative (0 points), weak (1 point), moderate (2 points), or strong (3 points). The staining field was defined as follows: less than 10% (0 points), 11–25% (1 point), 26–50% (2 points), and >50% (3 points). The obtained values were multiplied and scores of >4 points were considered as positive, whereas scores of <4 points were considered as negative. The stained area was measured using the ImageJ software (v.1.52a, National Institutes of Health, Bethesda, MD, USA) [10]. An example of the stain interpretation is shown in Figure 4.

#### 2.1.3. Statistical Analysis

Hierarchical cluster analysis was performed using the obtained data [11]. The chi-square test, Bonferroni correction, and residual analysis were used for the statistical analyses of the three subgroups (StatMate-III software, Atom, Tokyo, Japan). *p* < 0.05 was considered as the threshold for a statistically significant difference.

## 3. Results

### 3.1. Clinical Pathological Evaluation

The clinicopathological results of the 100 cases of early gastric neoplasia evaluated based on the WHO classification are shown in Table 1. In terms of the invasion depth, the frequency of T1a was higher in LGD (100%) and that of T1b was higher in IMA (25%) (*p* < 0.01) among the groups. In terms of the gross morphology, the elevated type was more frequent in LGD (70.8%), the mixed type was more frequent in HGD (11.1%), and the depressed type was more frequent in IMA (87.5%) (*p* < 0.01). The c-Myc positivity rate was higher in HGD (94.4%) and IMA (100%) compared with that in LGD (41.7%) (*p* < 0.01).

### 3.2. Hierarchical Cluster Analysis

**Figure 5:**
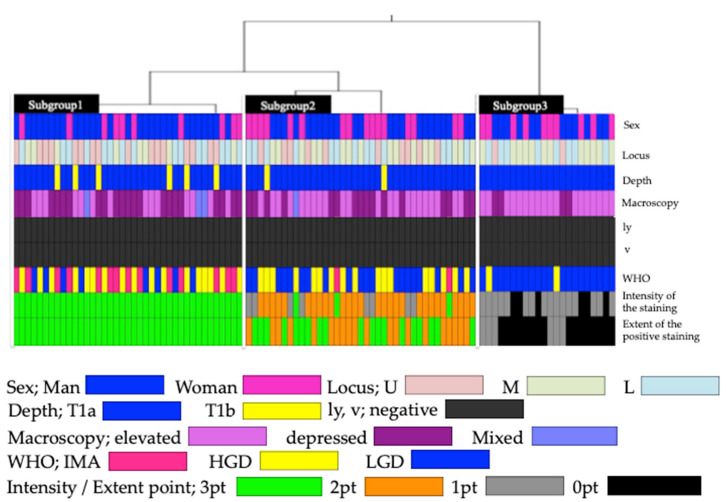
Hierarchical cluster analysis based on c-Myc expression. U: upper; M: middle; L: lower; ly: lymphatic invasion; v: venous invasion; LGD: low-grade dysplasia; HGD: high-grade dysplasia; IMA: intramucosal adenocarcinoma.

A hierarchical cluster analysis was performed based on the staining intensity, staining range, and score (Figure 5). Subgroups one, two, and three consisted of 39, 39, and 22 patients, respectively. Clinicopathological analyses were performed among the subgroups (Table 2).

An origin in the upper part of the body was significantly more frequent in subgroup one (41%) (*p* < 0.05), and the origin was proximal to the midline of the body significantly more frequently in subgroup three (54.5%) (*p* < 0.01). In terms of the invasion depth, the frequency of T1b was significantly higher in subgroup one (15.4%) (*p* < 0.05). In terms of the gross morphology, the elevated type was significantly more frequent in subgroup three (81.8%) (*p* < 0.01), and the depressed type was significantly more frequent in subgroup one (56.4%) (*p* < 0.05). 

The frequency of LGD was significantly higher in subgroup three (90.9%) than in subgroup one (20.5%) and subgroup two (51.3%) (*p* < 0.01). The frequency of IMA was significantly higher in subgroup one (35.9%) than in subgroup two (5.1%) and subgroup three (0%) (*p* < 0.01). The frequency of HGD was significantly lower in subgroup three (9.1%) than in subgroup one (43.6%) and subgroup two (43.6%).

The c-Myc positivity rate was significantly higher in subgroup one (100%) than in subgroup three (0%) (*p* < 0.01), while that in subgroup two (79.5%) did not differ significantly from that in the other groups.

## 4. Discussion

The molecular pathogenic mechanisms of cancer can be broadly classified into genomic and epigenomic abnormalities [12]. Genomic abnormalities include the loss of heterozygosity, mutations, and CNA. In recent years, many genes that may be key drivers of gastric cancer have been reported. In 2018, Nanki et al. [13] reported that most gastric cancers depend on the growth factor Wnt. Abnormal Wnt signaling induces the nuclear heteroaccumulation of β-catenin, which in turn induces abnormal cell proliferation via the overexpression of oncogenes, such as cyclin D1 and c-Myc. As a result of the CNA analysis of early gastric cancer, a gain of c-Myc was frequently observed, which may be closely related to abnormalities in Wnt signaling [3,4]. The current study was conducted to clarify the biological importance of c-Myc expression in early gastric neoplasia based on the WHO classification.

The cluster analysis was performed based on the c-Myc staining results. Each cluster showed independent clinicopathological features, which could be classified into three patterns in terms of c-Myc expression: subgroup one, characterized by a high c-Myc expression, high frequency of IMA, and depressed gross morphology; subgroup three, characterized by a low c-Myc expression, and most cases involving LGD and an elevated gross morphology; subgroup two, exhibiting intermediate characteristics between subgroups one and three, with no significant differences. Notably, the positive rate of c-Myc expression was 100% for IMA, 94.4% for HGD, and 41.7% for LGD. In early gastric neoplasia, c-Myc expression was correlated with nuclear and structural atypia. The incidence of immunostaining in early gastric cancer was reported as 18.1–100% [14,15,16,17,18,19,20]. However, these studies included both differentiated and poorly differentiated adenocarcinomas and were not evaluated using the WHO classification. 

Nakayama et al. [21] reported highly interesting data on c-Myc expression. They used laser microdissection to extract DNA from intramucosal carcinoma, submucosal invasive carcinoma, and advanced carcinoma, and performed a CNA analysis by array comparative genomic hybridization. Myc loss and TP53 gain are defined as dormant patterns, whereas Myc gain and/or TP53 loss are defined as aggressive patterns. The results of the genealogical analysis suggested that differentiated adenocarcinomas with dormant patterns rarely develop into advanced cancer. In addition, some intramucosal carcinomas showed an aggressive pattern. This disease state may have undergone an epigenetic change (methylation) that was, subsequently, corrected. The c-Myc expression rate of LGD in this study was 41.7%. LGD with c-Myc expression is referred to as aggressive LGD, and LGD without c-Myc expression is referred to as dormant LGD. Aggressive LGD may readily progress to HGD and IMA.

In gastric cancer, c-Myc expression is an indicator of malignancy and poor prognosis [14], but is not necessarily high in patients with advanced gastric cancer. The level of the c-Myc messenger RNA expression has been reported as higher in early gastric cancer than in advanced gastric cancer [22]. c-Myc has been shown to further increase the expression level of genes with some level of expression and to alter the characteristics of cancer cells [23,24]. Therefore, the expression of c-Myc is thought to be a genetic abnormality in the early stages of carcinogenesis.

Presently, there are no reports, including basic research studies, on the potential of c-Myc as a therapeutic target in advanced gastric cancer. This is because c-Myc is a nuclear molecule and has no target-binding site for small molecules, making it unsuitable for drug design [25]. In contrast, BET inhibitors (JQ1, ARV-825), which indirectly inhibit c-Myc, have been reported in hematopoietic tumors [26,27]. Further studies are required to determine whether BET inhibitors can be used to treat solid tumors, including gastric cancer.

## 5. Conclusions

We observed a clear clinicopathological difference in c-Myc expression in early gastric neoplasia based on the WHO classification. These results suggested that the dormant LGD tumor group belongs to a different category than aggressive LGD, HGD, and IMA. The expression of c-Myc is thought to be a key element in the early stages of carcinogenesis. When biopsies are taken by upper endoscopy and proliferative LGD is diagnosed, c-Myc staining can be used as a supplementary tool to determine whether the tumor is aggressive. However, gastric cancer is considered to have a strong heterogeneity and should be carefully evaluated.

## Figures and Tables

**Table 1:** Clinicopathological findings of early gastric neoplasia patients.

	LGD	HGD	IMA	*p* Value
**Total**	48	36	16	
**Age (range)**	78 (57–87)	79.5 (62–94)	78.5 (64–92)	N.S
**Sex (Man/Woman)**	30/18	22/14	12/4	N.S
**Locus**				
**Upper**	13	10	4	N.S
**Middle**	22	8	6	N.S
**Lower**	13	18	6	N.S
**Depth (%)**				
**T1a**	48 (100)	32 (88.9)	12 (75)	<0.01, N.S, <0.01
**T1b**	0 (0)	4 (11.1)	4 (25)	<0.01, N.S, <0.01
**Macroscopy (%)**				
**elevated**	34 (70.8)	18 (50)	2 (12.5)	<0.01, N.S, <0.01
**depressed**	14 (29.2)	14 (38.9)	14 (87.5)	<0.05, N.S, <0.01
**Mixed**	0 (0)	4 (11.1)	0 (0)	<0.05, <0.01, N.S
**c-Myc expression (%)**				
**positive**	20 (41.7)	34 (94.4)	16 (100)	<0.01, <0.01, <0.01
**negative**	28 (58.3)	2 (5.6)	0 (0)	<0.01, <0.01, <0.01

Low-grade dysplasia; LGD, high-grade dysplasia; HGD, intramucosal adenocarcinoma; IMA, not significant; N.S.

**Table 2:** Clinicopathological findings based on Hierarchical cluster analysis.

	Subgroup1	Subgroup2	Subgroup3	*p* Value
**Total**	39	39	22	
**Age (range)**	78 (63–93)	78 (57–94)	78 (68–88)	N.S
**Sex (Man/Woman)**	29/10	23/16	12/10	N.S
**Locus (%)**				
**Upper**	16 (41)	10 (25.6)	2 (9.1)	<0.05, N.S, <0.05
**Middle**	8 (20.5)	16 (41.0)	12 (54.5)	<0.01, N.S, <0.01
**Lower**	15 (38.5)	13 (33.3)	8 (36.4)	N.S, N.S, N.S
**Depth (%)**				
**T1a**	33 (84.6)	37 (94.9)	22 (100)	<0.05, N.S, N.S
**T1b**	6 (15.4)	2 (5.1)	0 (0)	<0.05, N.S, N.S
**Macroscopy (%)**				
**elevated**	14 (35.9)	22 (56.4)	18 (81.8)	<0.01, N.S, <0.01
**depressed**	22 (56.4)	16 (41.0)	4 (18.2)	<0.05, N.S, <0.05
**Mixed**	3 (7.7)	1 (2.6)	0 (0)	N.S, N.S, N.S
**WHO (%)**				
**IMA**	14 (35.9)	2 (5.1)	0 (0)	<0.01, <0.05, <0.05
**HGD**	17 (43.6)	17 (43.6)	2 (9.1)	N.S, N.S, <0.01
**LGD**	8 (20.5)	20 (51.3)	20 (90.9)	<0.01, N.S, <0.01
**c-Myc expression (%)**				
**positive**	39 (100)	31 (79.5)	0 (0)	<0.01, N.S, <0.01
**negative**	0 (0)	8 (20.5)	22 (100)	<0.01, N.S, <0.01

Low-grade dysplasia; LGD, high-grade dysplasia; HGD, intramucosal adenocarcinoma; IMA, not significant; N.S.

## Data Availability

The data that support the findings of this study are available from the corresponding author, N.A., upon reasonable request.
